# Perspectives and ethical considerations for return of genetics and genomics research results: a qualitative study of genomics researchers in Uganda

**DOI:** 10.1186/s12910-021-00724-1

**Published:** 2021-11-19

**Authors:** Joseph Ochieng, Betty Kwagala, John Barugahare, Erisa Mwaka, Deborah Ekusai-Sebatta, Joseph Ali, Nelson K. Sewankambo

**Affiliations:** 1 Department of Anatomy, College of Health Sciences, School of Biomedical Sciences, Makerere University, P.O Box 7072, Kampala, Uganda; 2 School of Business and Management Studies, Makerere University, Kampala, Uganda; 3 School of Humanities, Makerere University, Kampala, Uganda; 4 Johns Hopkins Bloomberg School of Public Health, Baltimore, USA; 5 Johns Hopkins Berman Institute of Bioethics, Baltimore, USA; 6 School of Medicine, Makerere University, Kampala, Uganda

**Keywords:** Perspectives, Ethical considerations, Return of results, Genetics, Genomics research, Researchers, Uganda

## Abstract

**Background:**

The return of genetics and genomics research results has been a subject of ongoing global debate. Such feedback is ethically desirable to update participants on research findings particularly those deemed clinically significant. Although there is limited literature, debate continues in African on what constitutes appropriate practice regarding the return of results for genetics and genomics research. This study explored perspectives and ethical considerations of Ugandan genomics researchers regarding the return of genetics and genomics research results.

**Methods:**

This was a qualitative study that employed in-depth interviews. Thirty participants were purposively selected based on their expertise as genomics researchers in Uganda. Data were analysed through content analysis along the main themes of the study using a comprehensive thematic matrix, to identify common patterns arising from the narratives. NVivo software 12 was used to support data analysis.

**Results:**

The return of genetics and genomics research results was generally acceptable to researchers, and some indicated that they had previously returned individual or aggregate results to participants and communities. The main reasons cited for sharing research results with participants included their clinical utility, actionability and overall benefit to society. Ethical considerations for appropriate return of results included a need for effective community engagement, genetic counselling prior to disclosure of the results, adequate informed consent, and proper assessment of the implications of, or consequences of returning of results. However, the approaches to return of results were perceived as unstandardized due to the lack of appropriate regulatory frameworks.

**Conclusions:**

The return of genetic and genomic research results is generally acceptable to researchers despite the lack of appropriate regulatory frameworks. Ethical considerations for return of genetics and genomics research results are highly divergent, hence the need for national ethical guidelines to appropriately regulate the practice.

**Supplementary Information:**

The online version contains supplementary material available at 10.1186/s12910-021-00724-1.

## Background

Genetics and genomics research (GGR) raises several ethical challenges both locally and international [1–5]. Issues concerning informed consent, privacy, confidentiality, risk and benefit analysis as well as community engagement are still unresolved, particularly regarding the return of genomics research results [6, 7].

Global debate on what constitutes socially acceptable and ethical approaches to sharing of GGR results, including incidental findings continues [8–22]. Feedback to research participants is an ethical requirement aimed at updating participants on study findings particularly those deemed clinically significant. However, it has been observed by the Human Health Heredity (H3Africa) network that there is limited guidance on the return of GGR results to research participants in Africa [19]. Most countries in sub-Saharan Africa lack appropriate regulatory frameworks for GGR [8, 9, 23]. Several international guidelines have been developed for the ethical conduct of GGR however these frameworks are generic and need to be contextualized to the local settings where such research is conducted [18, 19]. Ethical conduct of GGR requires clear understanding of the culture, traditions, and perceptions of the local research community; however, this data is largely missing, especially from sub-Saharan Africa. This is especially true about the return of GGR results to participants and communities. Generally, there is limited data on people’s preferences and perspectives that is critical for the contextualization of GGR processes [8, 9]. In a study conducted in Uganda, Rutakumwa et al. [6] reported that participants of genomic research were very much interested in the clarity of the nature and timing of return of genomic research findings. There is thus need for stakeholder engagement as we ponder on how to enhance the current national ethics guidelines in Uganda to address weaknesses regarding GGR [24].

This study, therefore, set out to explore the perceptions, experiences, and ethical considerations of genomics researchers regarding the return of GGR results to inform the enhancing of national ethics guidelines that is needed to ensure standardization of the relevant practices in the country.

## Methods

This was a cross-sectional study that employed a qualitative exploratory approach. The study was conducted by a team of academics comprising of social scientists, bioethicists, and medical scientists with experience in qualitative research. JO a male MD academician with bioethics training and experience and BK a female PhD sociology academic of more than 20 years led most of the interviews. They were assisted by bioethics master’s graduates with gender consideration. Data were collected between July 2019 and September 2020 among researchers who were actively involved in GGR. Respondents were recruited from institutions in the central, western, eastern, and northern regions of Uganda. Most of the participants were colleagues in research and academia and were well known to the researchers; but the two parties had no close working relationships. Data were collected using in-depth interviews that were guided by a semi-structured interview schedule adapted from the informed consent checklist for genetic and genomic testing of the USA Federal Code of Regulations [25], the USA regulations for the protection of human subjects in research at 45CFR 46 which provides detailed information on the requirements for research participants to be respected throughout the research process [26]. The interview guide is attached as an Additional file: 1. Questions, prompts and guides were provided by the interviewers. Thirty researchers were purposively selected based on their expertise as principal investigators on GGR projects and were identified with the help of a leading molecular immunologist and GGR researcher. The identified individuals were approached either in person, by telephone or via emails and requested for a convenient appointment. All the individuals approached accepted to participate in the study.

Face to face in-depth interviews were privately conducted at the respective offices of the respondent and lasted about 40–70 min. All interviews were conducted in English. The main domains covered in the interviews included information about respondents’ perceptions, experience, and practices on GGR and the return of results, procedures involved in their respective studies and the ethical guidance available to them for decision making. Responses were audio recorded and complemented by field notes taken by research assistants. All audio recordings were transcribed verbatim and checked for accuracy before analysis. Data were analysed through content analysis along the main themes of the study. A comprehensive thematic matrix that included codes, categories, and themes to identify common patterns arising from the narratives, was developed. The coding was done both deductively and inductively. Transcripts were further reviewed for emerging themes that were integrated into the thematic matrix. Multiple people JO, BK, DES were involved in applying and confirming application of codes across all transcripts and disagreements were resolved by cross checking with the recorded data. NVivo software (QSR international 2020) was used to support data analysis and illustrative quotes were extracted.

### Ethical considerations

Ethics approval was obtained from the Makerere University School of Biomedical Sciences Higher Degrees and Research Ethics Committee (ref. SBS-628) and Uganda National Council for Science and Technology (UNCST) (ref. SS268ES). Written informed consent was obtained from all respondents (both male and female) before enrolment in the study. All the methods were carried out in accordance with relevant national and international guidelines and regulations. No participant identifying information was recorded.

## Results

Most participants were male (23/30) with a mean age of 41 years (range 29–65 years). All were residents of Uganda and affiliated to at least one of research/academic institution in the central, western, eastern, or northern regions of the country. Most respondents were researchers and academicians as summarized in Table 1.

**Table 1:** Participant characteristics

Attribute	No of participants N = 30	Male	Female
*Age range*			
Missing age	1		1
20–29	1	1	0
30–39	10	8	2
40–49	10	8	2
50–59	6	6	0
60–69	2	0	2
*Highest level of education attained*			
Masters	9	8	1
PhD	21	15	6
*Employment/position*			
Researcher	6	5	1
Lab associate	3	3	0
Dean	2	1	1
Lecturer	11	10	1
Professor	4	2	2
Senior scientist	1	0	1
Director	2	1	1
Graduate fellow	1	1	0
*Duration/experience in genomics research*			
1–4 years	6	5	1
5–10 years	15	13	2
11–15 years	5	3	2
> 15 years	4	2	2

Many of the participants held multiple positions

Respondents’ field of specialization included molecular biology, immunology, microbiology, biochemistry, pharmacology, internal medicine, transfusion medicine, surgery and obstetrics and gynaecology. Type of studies conducted by respondent included molecular diagnostics, pharmaco-genetics, pharmaco-genomics, molecular genotyping, microbio-genotyping and haematological genomics.

Three themes emerged from the data.

1. On perceptions, sharing of genetics and genomics research findings was acceptable and one theme emerged with two sub-themes.
2. On ethical considerations, one theme emerged with three sub-themes.
3. On experiences, one theme emerged with one sub-theme Table 2.

**Table 2:** Themes and sub-themes

Objective	Theme	Sub-theme
Perceptions	Perceptions to feedback of results	(i) Barriers to return of results (ii) Role of formal guidance
Ethical considerations	Appropriate communication of research results	(i) Community engagement (ii) Genetic counselling (iii) Informed consent and implications
Experience	Experience and practices on feedback of results	(i) Challenges to return of results

### Perceptions to feedback of results

Most respondents supported the return of individual and aggregate genetic and genomic research results. Respondents noted that there is an ethical imperative to share research findings with participants and communities to not only satisfy their curiosity but also as a benefit, particularly if the findings are clinically significant and actionable. Respondents further reiterated the importance of sharing GGR results that are of public health importance; they felt that such results were invaluable in disease prevention and surveillance.

I think it is because when there is a finding the owner of the results has that right to know to get that information. R004.

Other respondents thought that it is good to share genetics and genomics results if such findings are likely to lead to public health interventions. They felt that if it is actionable or of public health importance then results should be shared because no individual wants to suffer from a disease if it can be prevented or cured following early diagnosis.

If it really has a big implication on public health that needs to be reported, but sometimes it may not have a direct benefit. So, you weigh the risks and benefits. R022.

Some respondents opined that failure to communicate findings that are potentially beneficial is tantamount to violation of the ethical principles of beneficence.

Do no harm is one of the cardinal principles of ethics, if you’re doing a study and one of the outcomes of the study is showing something that could affect the patient, you’re obliged to give the results. R013.

The issue of confidentiality in the return of results was emphasized because respondents felt that a breach in confidentiality can have far reaching psychological and social implications, as one researcher stated.

You need to tell only those who are affected because issues like stigmatization come up. If you say the following ten have this disease, you are creating problems for those people because they will be segregated. R014.

Not all respondents favoured the sharing of GGR findings with participants. They pointed out that most of the genomic analyses are performed abroad and were not primarily meant for diagnostic purposes. Respondents also felt that many research participants might not have the capacity to comprehend the results at hand. They further asserted that some of the results might not be good, and yet many research team members are not well trained to communicate such sensitive information. They indicated that this challenge could be solved by genetic counsellors, who unfortunately are lacking in the country.

We could have but we didn’t. it is very scientific that some of those things don’t make sense to even scientists. R027.

### Barriers to return of results

Respondents highlighted several factors that impede the return of GGR results. They posited that Uganda does not have experts who can analyse and accurately interpret these results. In additions, they argued that most of the results make no sense and require very specialized analysis. Respondents further indicated that translating of the results into local languages to enhance comprehension could also be problematic, since many of the research participants are illiterate. Some respondents conceded that they had never thought of returning GGR results because of lack of national guidance on how this should be done.

They highlighted the need for appreciation and evaluation of the implications of the shared results such as stigma, discrimination, litigation, or family breakups that may affect the individual research participant, their family members, or their entire community. Others observed that identifying one genetic condition may not be enough for predicting development of a disease because genetics do not always lead to Phenotypic presentation.

And these issues I think they have legal implications…… you really have to rethink and then probably get advice on how best to handle these things like you have said context matters. R009.

### Role of formal guidance

Respondents pointed out the lack of national and institutional guidance as one of the major challenges to the return of GGR results in Uganda. They argued that proper oversight, based on a contextualized regulatory framework is vital for ethical return of GGR results, as stated in this observation.

We need to have some guidelines and polices in place especially for clinical research or studies that go deep to look at the genetics of individuals in a hope of coming up with better treatment options for these people. R002.

### Appropriate communication of research results

Respondents observed that if sharing of GGR results with participants is to be meaningful and appropriate, several ethical considerations are necessary to prepare both the participants and the researchers.

### Community engagement

Most respondents emphasized the importance of community engagement in preparing individuals, families, and entire communities to receive GGR results. This position stemmed from the perceived implications of GGR results that could potentially extend beyond the individual to involve the community. This they stressed, should be considered right from the inception of the study. Respondents also gave several suggestions on community engagement approaches including talking to community leaders, holding community meetings, radio talk shows with call-ins, use of Community Advisory Boards (CABs) and so on.

When we needed to know the burden of disease in the community, we first told them what the protocol was about and then how the community would contribute. This is particularly when we wanted to take samples that are community based to compare with samples which are hospital-based surveys. We’ve done this and even collected samples. R024.

### Genetic counselling

Respondents observed that, for return of GGR results to be meaningful, safe, and effective, there is need for appropriate genetic counselling by qualified individuals who can accurately explain the meaning and implications of both participation in GGR as well as associated results.

Yeah, there should be someone who is at least knowledgeable who understands the terms and who can explain in simple terms the implications of such studies and what happens if indeed an individual, a family or a community are carrying a certain gene that may not be very good in the eyes of the public so there should be this person who can talk to people. R003.

Respondents observed that for medical genetics there is a need for all sorts of counsellors; the counsellors that prepare participants, clinicians, and the researchers themselves. They stressed the need for a counsellor who understands genomics and understands the implications of the kind of research to be conducted.

Very very important, because of the possible outcomes, we should have genetic counsellors. Whenever you talk about genetics first of all there is a lot of misunderstandings, so presence of genetic counsellors would help us sort out those possible misunderstandings. R005.

Some respondents who indicated that they had returned GGR result reported that they used ordinary nurse counsellors to do the genetic counselling. None of the respondents had genetic counsellors on their projects.

The participants who come through our research program go through a very long process of counselling. For instance, one of our studies, we first of all take two months and during that time we do counselling, and we give them opportunity to ask questions. After the two months we do the decisive tests and by the time we give them the results, it’s much easier. R024.

I would say they are specialized through practice not through training. So, they have gone through the protocol and the standard procedures for counseling, but they have no general training on genetic counseling. R030.

### Informed consent and implications on the return of results

To increase the ethical acceptability for feedback of individual and aggregate GGR results, it was deemed appropriate to obtain informed consent from participants. Informed consent was seen as a process of giving participants adequate relevant information about the study; the potential risks, particularly social harm to the family and at times, the community; and enhancing participant comprehension using simple language and visual aids such as videos.

Of course, you have to go back looking through the consent. If you find something that is really important and the patient consented, you have to invite the patient back. Literally in a normal context this is supposed to be a genetic counsellor to give this information and genetic counselling…. R010.

Respondents stressed the role of the researcher in making appropriate decisions especially regarding the utility of the findings and favorable risk–benefit assessment.

In terms of feedback, you have to be very sensitive to the implications of your results to the participant and to the community. So, you have the information, and you have the discretion to judge. That is your discretion as a researcher. R019.

Much as all respondents concurred that informed consent was an ethical imperative, some of them conceded that they did not provide adequate information on how GGR results would be handled.

We explain to them the study and what the study is going to do but we don’t tell them that we’re coming back to share what we find in their genes. R008.

### Experience and practices

Some respondents reported that they had ever returned either individual or aggregate GGR results to participants and their communities. They indicated that test results were either shared with individual participants or through attending clinicians. At least three approaches were used to communicate research results including individual counselling followed by sharing of results by researchers themselves; submitting results to the attending clinicians for the necessary communication to respective individual study participants; or, in the case of aggregate results, through community meetings as highlighted below.

What I know is that for us we were giving individual results…. fortunately, the results were good, people were excited, and they had to go and tell everyone that you know what this has potential… R005.

Yes, for instance we typed over 2000 community samples for 3 conditions, sickle cell disease, beta-thalassemia and glucose-6-phosphate dehydrogenase and those that we return, we return all the results as individual results and those that we found positive, we’ve been able to refer them to specialized treatment centres. R024.

It was also observed that the amount of information provided to research participants varied across studies with some giving definitive test results while others shared just bits of the genetic information. Other aspect included how the research participants used their genetics and genomics research results particularly when seeking health care.

Yes, we have to give them some information of what we found. Why I say some information? Explaining genetics to anybody is complex, but we have to go back to the participants and give them, not individual, but as a group. So, we don’t give individual responses. R026.

Respondents also shared their experiences on community engagement during the conduct of GGR. Some respondents developed and used videos to explain genes based on the concept of a cell as a building block of the body.

We realized the leadership within the community is very important, so the consent was just not limited to the individual subjects in the study, we had to move on to the leadership. Yes, I agree, maybe community consent might be of use in the context of this kind of thing. R010.

### Challenges to return of results

Several GGR studies did not return findings to participants for various reasons including the inability to trace participants because samples were de-identified; no prior intention of offering feedback to participants because the genomic/genetic analysis was a secondary study; and most results had no clinical utility.

No, but us we don't report directly to individuals because in all genetic studies, we have to de-identify the data, we cannot trace back the individuals, it’s one of the ethical aspects of this, we work with de-identified data. We can’t report directly to individuals, but we can report to the community. Yes, maybe aggregated results but nothing personal. R011.

Respondent highlighted challenges encountered regarding the return of GGR results including the lack of guidelines and regulations on what constitutes appropriate practice.

I don’t think there is a particular procedure other than riding on the fact that we had informed the REC that we shall disseminate findings. So, we just went back to the hospital, which was the base and, maybe the other thing done was we spoke with the in-charge of the children’s ward and the one who was in charge of the Outpatients Department of the paediatrics unit… but we didn’t follow any procedures or follow up participants. R003.

## Discussion

The study set out to explore researchers’ perceptions, experiences, and ethical considerations for return of GGR results.

Our study results show that return of GGR results is generally acceptable among researchers. Several challenges were identified including interpretation of what is beneficial or clinically significant, meaning of the findings, understanding of GGR terminologies by the participants and the lack of a context specific ethical and regulatory framework.

Ethical issues surrounding the return of GGR results including the extent to which such results should be shared with participants have been extensively discussed globally [10–17]; however, there is dearth of literature from sub-Saharan Africa. While international policies for return of individual genetic research findings are still evolving, there seems to be growing consensus on the necessity for returning GGR findings to participants [18, 19]. The return of GGR results to participants is a complex process that follows stringent internationally accepted criteria to ensure that only credible and verified findings are communicated [19, 20]. The H3Africa Consortium has come up with guidelines for the return of GGR results in Africa that include a decision tree [19]. The criteria set forth in these guidelines (1) methods used to generate those findings should be able to detect genetic variant(s) accurately and reliably in the affected individual, (2) genetic variant (s) should be robustly associated with disease causation, thereby accurately and reliably predict clinical outcome and (3) findings should be able to guide therapy or prevent disease and/or have proven therapeutic or preventive intervention. In addition, there should be some indication that participants wish to receive findings [9]. Although GGR in Uganda is steadily increasing, public debate on this issue is limited, we only found three articles from Uganda [6, 21, 27].

A few researchers in this study reported having returned GGR result before while a majority had never done so. Those that had returned results reported preparing participants and communities prior to offering any feedback. This is the recommended practice that has been widely used elsewhere to reduce on the negative impact GGR results could have on the community [20, 22]. Our findings suggest that there is a weakness in the decision-making process regarding the return of GGR results. The few who had returned GGR results before indicated that the decision and how to return results was at the discretion of the researcher. This was majorly attributed to the absence of appropriate regulatory frameworks for GGR in Uganda as well as lack of awareness of the available international guidelines. Our view is that without such standards to provide a basis for regulation, it will remain difficult to ensure ethical accountability in GGR in Uganda. Therefore, to minimize the arbitrariness of important decision making, there is need for specific local regulatory frameworks for this undertaking. We believe that the findings of this study will go a long way in contributing to the literature and data for development of context specific ethical guidelines for the conduct of GGR in Uganda.

Community engagement was considered an important requirement for the return of GGR results. The need for meaningful community engagement is increasingly being promoted in global health research with the aim of protecting communities from exploitation and harm while promoting research that is beneficial [28]. Community Advisory Boards have been employed mainly by clinical trials but different methods could be used based on the nature and setting of the study [29, 30]. Community engagement is essential if communities are to understand the ethical and social implications of the study particularly the findings. There is need to consider and respect local culture, traditions, social values and preferences [30]. This is particularly important because the Ugandan society is quite diverse in terms of traditional, cultural, and social aspects.

Our results suggest that genetic counselling is fundamental and a requisite to the return of GGR results, but there is a major weakness in Uganda because the necessary cadre of professionals is lacking. Genetic counsellors are essential in ensuring that research participants adequately understand what the study entails and the implications of their participation [1, 31–34]. Thus, there is need for human capacity development for the ethical conduct of GGR.

Informed consent is the mainstay of ethical research; therefore, participants should be given adequate relevant information, including on how results will be handled. Unfortunately, most respondents indicated that they did not discuss the issue of results with participants. This is a requirement in the current Ugandan ethics guidelines however, implementation and oversight are poor. This suggests the need for considerable attention and appropriate oversight by the research ethics committees (RECs) based on contextualized guidelines [35, 36]. Such oversight would help researchers appreciate the informed consent process and enhance areas that have hitherto been ignored. The need for feedback of GGR results has been considered by research participants in Botswana as a form of solidarity and reciprocity obligations of researchers and, can make participants feel valued as part of a mutual relationship [37]. Similarly, the need for feedback of GGR results has been documented in related work among genomic research participants in Uganda [27]. Although the Botswana and Uganda studies involved genomics research participants while this study involved researchers, the message is quite clear across all the studies that feedback of GGR results is necessary.

The Inappropriate return of GGR results has potential for breach of confidentiality and associated harms such as psychological harm, stigmatization and family conflict for individual research participants, their families or the entire community [38, 39]. Other harms like denial of insurance or increased premiums and loss of income may occur as well. Hence the need for the Ugandan contextualized ethical guidelines with clear measures on how participants’ genetics information is to be protected from unauthorized access and misuse. Additionally, participants should be given an opportunity to decide whether they would like to receive the results, mode of return of results and what type of results they want to receive [1, 40–43]. Findings of this study highlight a need for standardization of practices for the return of GGR results through appropriate oversight by RECs based on a contextualized ethical framework or guidelines.

### Limitations

We acknowledge that there could have been a potential for social desirability bias that could make respondents to report favouring what they think to be societally preferred under the circumstances [44].

The study reported in this manuscript involved only researchers yet perceptions of other key stakeholders like research participants and research regulators would have provided a more complete picture on the views and experiences regarding genetics and genomics research findings with participants. However, there is some literature concerning GGR research participants in the Ugandan setting [27]. Additionally, similar work involving various research stakeholders is currently on going by the same research team which we hope will enrich the available data for guideline development.

Findings reported in this manuscript were generated using a qualitative approach and limits the generalizability of the findings. However, related work employing quantitative approached is underway to address the issue of generalizability of the data.

Finally, many respondents were known to the researchers, and this might have put them in a situation where they felt obliged to participate.

## Conclusions

Feedback of GGR research results to participants is generally acceptable to genomics researchers and several researchers have returned either individual or aggregate results. Ethical considerations for return of GGR results are numerous though their application is not regulated due to lack of appropriate local ethical guidelines.

## Supplementary Information

**Additional file 1.** In-depth Interview guide.

## Data Availability

Data sources are available on request. Request can be made to the corresponding author at ochiengjoe@yahoo.com.

## Abbreviations

CABs: Community Advisory Boards
GGR: Genetics and genomics research
REC: Research Ethics Committee
SOPs: Standard Operating Procedures
UNCST: Uganda National Council for Science and Technology
