# Supplementary material for: Perspectives and ethical considerations for return of genetics and genomics research results: a qualitative study of genomics researchers in Uganda
Source: BMC Med Ethics. 2021 Nov 19;22:154. doi: 10.1186/s12910-021-00724-1 (PMC8603565; doi:10.1186/s12910-021-00724-1)
Supplement: Supplementary file 1 — Additional file 1. In-depth Interview guide. [file 12910_2021_724_MOESM1_ESM.docx]

**APPENDIX 3**

**Title: Ethical Legal and Social issues associated with return of results for Genetics and genomics research in Uganda**

**GGR Tool for IDIs with Researchers in genetics and genomics**

**Background information**

I am going to ask you some questions about your background. This information helps us to know about our respondents (your identity will remain confidential) *(could be self-administered if separate from the tool)*

|  |  |  |
| --- | --- | --- |
|  | Sex (observe) | 1. Male 2. Female 3. Other |
|  | Place of work/ institution | ……………………………………………………………………… |
|  | Occupation/position | ……………………………………………………………………… |
|  | Field of specialization | ……………………………………………………………………… |
|  | Highest level of education attained | 1. Bachelors 2. Masters 3. PhD   Other (specify) ……………………………………………………………………… |
|  | Duration of participation in GGR research | ……………………………………………………………………… |
|  | How old are you? | ………………… Years  Prefer not to say |

**We would like you to share with us about your experience in conducting genetics and genomics testing**

**A: Experience:**

1. What is/was the nature of genetics and genomics study(ies) you have conducted? Did any of your studies involve sample collection? *(if so*) What special genetic information about your participants are/were you interested in? For what purpose(s) did/are you collecting the samples? *(Ask for each of the studies if more than one).*
2. What benefits do/did you anticipate for your participants as individuals, families and communities in which you conducted (or are conducting) the study?
3. Is/was any of your study(ies) of such a nature that you had to report back to your participants about what you had discovered about their genetic makeup (*and what that means for their physical health and social lives as individuals, families of whole communities*)? What procedures does/did the research team follow? What guided your practice? What is/was the outcome (so far)?

Is it necessary to return results? Have you ever returned results? *(if yes*) Under what conditions did/do you return results in genetics and genomics research? Did you follow any guidelines? When would it necessary to return aggregate results? What about individual results?

In case of incidental findings, how do you handle such results*? (if respondent had such result)* Do you return or communicate them to participants? What guides your practice? Under what conditions to you return such results? (*If none yet ask how they would handle in case of such results)*

Do/did you have any specialized genetic counsellor on your research team of any of your genetics and genomics studies? Why/Why not?

**B: Attitudes**

1. Following genetics testing, should results of genetics and genomics testing be shared? With whom? Patient? Family? Community?
2. Why would results of genetics and genomics testing be shared? Anticipated benefit, risk?
3. How should results of genetics and genomics testing be shared? (Ethical review and approval, Informed consent, Genetic counselling, community engagement? Who should share the results immediate family, blood relative, community and beyond?)
4. What are the implications of sharing genetics and genomics results?
5. How about incidental findings? In case of incidental findings, how do you handle such results*? (if respondent had such result)* Do you return or communicate them to participants? Under what conditions to you return such results? (*If none yet ask how they would handle in case of such results)*
6. Do/did you have any specialized genetic counsellors on your research team of any of your genetics and genomics studies? Why/Why not?
7. How should feedback of results be carried out? Any guidelines? How about incidental findings?
8. What else would you like to share about the experiences and challenges for return of results during genetics and genomics testing?

Thank you for participating in the study.
